# Workload assessment: cross-cultural adaptation, content validity and instrument reliability

**DOI:** 10.1590/0034-7167-2022-0556

**Published:** 2023-08-07

**Authors:** Caroline Lopes Ciofi-Silva, Luciana Cordeiro, Naila Albertina Oliveira, Giulia Marcelino Mainardi, Anna Sara Levin, Rodrigo Maximiano Antunes de Almeida, Juliana Falasco-Fantinatti, Maria Clara Padoveze

**Affiliations:** I Universidade Estadual de Campinas. Campinas, São Paulo, Brazil; II Universidade Federal de Pelotas. Pelotas, Rio Grande do Sul, Brazil; III Universidade de São Paulo. São Paulo, São Paulo, Brazil; IV Universidade Federal de São Paulo. São Paulo, São Paulo, Brazil; V Universidade Federal de Itajubá. Itajubá, Minas Gerais, Brazil; VI Hospital Municipal de Paulínia. Paulínia, São Paulo, Brazil

**Keywords:** Workload, Translating, Validation Study, Health Personnel, Work, Carga de Trabajo, Traducción, Estudio de Validación, Personal de Salud, Condiciones de Trabajo, Carga de Trabalho, Tradução, Estudo de Validação, Pessoal de Saúde, Condições de Trabalho

## Abstract

**Objectives:**

to adapt, validate the content and assess the reliability of the instrument National Aeronautics and Space Administration – Task Load Index, translated into Brazilian Portuguese.

**Methods:**

a methodological study, divided into five steps: translation; synthesis; back-translation; assessment of the Portuguese version by an expert committee; pre-test and content validity of the final version by health professionals working in inpatient units. The Content Validity Index (CVI) (minimum 0.80) and Cronbach’s alpha (minimum 0.70) were calculated.

**Results:**

in the first round, in the agreement analysis of the translated version, three items did not reach the minimum CVI value. It was decided to remove the statement. The instrument title and items “performance” and “effort” were changed. There was consensus and approval of the final version in the pre-test step.

**Conclusions:**

the NASA Task Load Index instrument, adapted to Brazilian Portuguese, presents reliability and content validity evidence.

## RESUMO

**Objetivos:**

adaptar, validar o conteúdo e avaliar a confiabilidade do instrumento *National Aeronautics and Space Administration – Task Load Index* , traduzido para o português brasileiro.

**Métodos:**

estudo metodológico, dividido em cinco etapas: tradução; síntese; retrotradução; avaliação da versão em português por comitê de especialistas; pré-teste e validação de conteúdo da versão final por profissionais de saúde atuantes em unidades de internação. Foram calculados o Índice de Validade de Conteúdo (IVC) (mínimo 0,80) e o alfa de Cronbach (mínimo 0,70).

**Resultados:**

na primeira rodada, na análise de concordância da versão traduzida, três itens não alcançaram o valor mínimo do IVC. Optou-se pela remoção do enunciado. O título do instrumento e os itens “desempenho” e “esforço” foram alterados. Houve consenso e aprovação da versão final na etapa de pré-teste.

**Conclusões:**

o instrumento Índice NASA de carga de tarefa, adaptado para o português brasileiro, apresenta evidências de confiabilidade e validade de conteúdo.

## RESUMEN

**Objetivos:**

adaptar, validar el contenido y evaluar la confiabilidad del instrumento *National Aeronautics and Space Administration – Task Load Index* , traducido al portugués brasileño.

**Métodos:**

estudio metodológico, dividido en cinco etapas: traducción; síntesis; traducción inversa; evaluación de la versión portuguesa por un comité de expertos; pretest y validación de contenido de la versión final por profesionales de la salud que actúan en unidades de hospitalización. Se calculó el Índice de Validez de Contenido (IVC) (mínimo 0,80) y el alfa de Cronbach (mínimo 0,70).

**Resultados:**

en la primera ronda, en el análisis de concordancia de la versión traducida, tres ítems no alcanzaron el valor mínimo de IVC. Se decidió eliminar la declaración. Se modificó el título del instrumento y los ítems “desempeño” y “esfuerzo”. Hubo consenso y aprobación de la versión final en la etapa de pre-prueba.

**Conclusiones:**

el instrumento *NASA Task Load Index* , adaptado al portugués brasileño, presenta evidencias de confiabilidad y validez de contenido.

## INTRODUCTION

The universe of work has been studied for hundreds of years, and knowledge about workload, both in relation to the definition of construct and its impact on workers’ psychodynamics, has been accumulated in recent decades. The process of globalization and changes in the world of work, considering the technological revolution and the entry of foreign capital into the economy, bring even greater complexity to this field^( 1 )^.

The new forms and relationships of work have aggravated the problems resulting from it and strongly impacted workers physically and psychologically^( 1 )^. Thus, the discussion about workload, a complex and polysemic term, and its measurement possibilities, remains current throughout the planet.

Workload is a broad and multifaceted term that encompasses the “cost” of performing a task by a human operator (e.g., fatigue, stress, illness, accidents, etc.)^( 2 )^. Hart and Staveland (1988), considering both the way in which people formulated opinions about workload and the subjective expression of the question, isolated and defined relevant factors for assessing this construct^( 2 , 3 )^.

Most scales that propose to measure workload or task load consider the respondents’ subjectivity, being necessary to correct the high variability of workers’ responses; another problem would be the variation in workload, depending on the task performed and the area of professional activity. Therefore, an index that considers the phenomenon’s multidimensionality and, at the same time, reduces subjective variability, in order to compute the global workload, was proposed^( 3 )^.

Currently, the National Aeronautics and Space Administration-Task Load Index (NASA-TLX) is a public domain index. Originally developed in English and initially aimed at the aviation field, the index assesses workload during or after performing a task. NASA-TLX can be applied during or immediately after completing a task or set of tasks, proposing to assess the performance in six dimensions, in order to determine a task’s global workload. Three of the six dimensions refer to aspects or requirements imposed by the subject (mental, physical and temporal), and the other three, to subject-task interaction (effort, performance and frustration)^( 3 )^.

As for the six dimensions of NASA-TLX, the “mental demand” dimension concerns the reasoning, decision-making or calculation required to perform a task; the “physical demand” dimension measures the amount and intensity of physical activity that is required to perform a task; the “temporal demand” is related to the amount of time involved to complete a task; the “effort” dimension assesses how hard workers need to make an effort to perform a task; the “performance” dimension concerns the performance of a task satisfactorily; and, finally, the “frustration” dimension assesses participants’ feelings while performing a task^( 3 )^. The response scale is presented by means of a horizontal line, divided into vertical lines in 20 equal intervals, without numerical demarcations, with bipolar ends, ranging from zero (low workload) to 100 (high workload). It is recommended to mark the desired location for each dimension, based on the experience of the task performed. The result is obtained by analyzing the corresponding point and adding increments of 5 to each demarcation. As for interpretation of results, the scores can be obtained from each dimension or the mean can be calculated, obtaining the global workload. So far, there is no strong evidence of reference values and score classifications. However, it is suggested that comparisons of the instrument results related to similar tasks be carried out^( 3 , 4 )^.

NASA-TLX is one of the most used indices worldwide to measure workload^( 4 )^. Twenty years after its development, it has been translated into more than a dozen languages, applied in different fields, and modified in many ways^( 2 )^. An online version for administering the index^( 5 )^ and software to simplify data collection, processing and storage processes^( 6 )^ were also created. The index was submitted to assessments in terms of reliability and sensitivity^( 7 )^, being compared with other workload measurement instruments^( 7 , 8 )^.

Brazilian researches have measured workload using mainly instruments that detect mental suffering, assess quality of life or measure psychosocial risk factors at work^( 9 )^. NASA-TLX has been used in Brazil in investigations in different contexts, with emphasis on industry, undergraduate and graduate students and health professionals^( 10 )^. In the international context, this index was also used in a study with clinical simulation to assess personal protective equipment (PPE)^( 11 )^.

The context of the coronavirus pandemic raised important questions about health professionals’ work processes and health conditions considered essential workers. It adds to the chronic problems of Brazilian public health, such as the under-funding of the Unified Health System (SUS – *Sistema Único de Saúde* ), the freezing of expenses in the sector, the deterioration of services, the precariousness of working conditions and the emerging challenges. Problems related to work management and staff training considering the opening of new beds in high complexity required work process reorganization, bringing new demands to workers. In this regard, measuring workload can be especially useful in this context, in order to propose actions to minimize problems arising from work^( 12 )^.

Although already used in Brazil^( 10 , 13 )^, there is no evidence of cross-cultural adaptation and validity for Brazilian Portuguese of NASA-TLX. Based on this context, there is a risk of translations and modifications of scales developed in a foreign language without in-depth study of their validity and accuracy of assessments carried out, which could compromise the equivalence between the original scale and the translated version and, consequently, their characteristics and assessed domains. Therefore, using a rigorous method of cross-cultural adaptation is necessary before applying an instrument developed in another language and culture^( 14 )^.

## OBJECTIVES

To perform the cross-cultural adaptation, content validity and reliability assessment of NASA-TLX into Brazilian Portuguese, with a view to ensuring that the index is suitable for application in studies in Brazil in different areas of professional practice.

## METHODS

### Ethical aspects

The study was conducted in accordance with national and international ethics guidelines, and was approved by the Research Ethics Committee of the Nursing School of University of Sao Paulo, whose appraisal is attached to this submission. The Informed Consent Form was signed by all individuals involved in the study: online, for experts, and in writing, for health professionals.

### Study design

This is a methodological study of translation and cross-cultural adaptation. Contact was made via email with the authors of the original version, requesting authorization to carry out cross-cultural adaptation. The authors informed that, as it is in the public domain, modifications and translations are authorized.

### Study protocol

**Figure 1:**
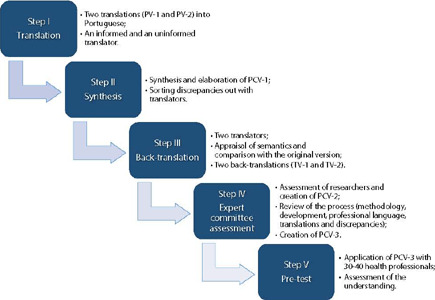
Flowchart of the cross-cultural adaptation process and validity of NASA-TLX

Source: adapted from Beaton et al., 2000.

The cross-cultural adaptation and content validity process was based on the method proposed by Beaton and on other studies of cross-cultural adaptation of psychometric instruments carried out in the Brazilian context^( 14 , 15 , 16 , 17 )^. The methodological steps were divided into five steps, illustrated in Figure 1 and detailed below:

I. Translation into Portuguese: performed by two bilingual translators, working at companies certified in translation, independently, with only one of the translators having knowledge of the objective of the instrument (informed translator), resulting in two versions in Portuguese (PV-1 and PV-2);
II. Synthesis of translation and elaboration of a consensual version in Portuguese: carried out by a third translator who, in a neutral way, produced a report demonstrating the means used to sort out divergences. Differences were analyzed and discussed between the three translators, resulting in the consensus version in Portuguese (PCV-1);
III. Back-translation: two translators, certified in English, translated the consensus version from Portuguese to English, resulting in two new versions (TV-1 and TV-2). These translators did not have access to the original version and concepts addressed;
IV. Expert committee assessment: the versions were obtained after back-translation; the main researchers of the project elaborated a new consensual version in Portuguese (PCV-2). This version, along with the others (original, PV-1, PV-2, PCV-1, TV-1, TV-2), was placed on a board and sent for assessment by an expert committee. This committee was composed of experts holding at least a master’s degree, fluent in English, from different areas of knowledge and activity, in order to obtain a multidisciplinary assessment of the process. Moreover, professionals working in the five regions of Brazil were invited, aiming to sort out possible cultural disparities in the translation process. Experts were selected through analysis of their *Curriculum Lattes* , using convenience sampling, and were invited to participate by email. After accepting the invitation, experts were instructed to record their opinions, answering a four-point Likert scale^( 18 )^ (“totally agree”, “agree”, “disagree” and “totally disagree”), if they agreed or not in relation to each item’s semantic, idiomatic, conceptual and cultural equivalence in the translated version compared to the original. Additionally, for each item, there was a field for them to write suggestions for changes or comments. Based on the Delphi technique^( 19 )^, the consensus version was obtained, named PCV-3^( 18 , 19 )^;
V. Pre-test: PCV-3 was applied to health professionals from a public hospital in a city in the countryside of Sao Paulo. Professionals with experience in assisting patients in hospital for at least six months were included. Professionals with some physical or psychological restriction for work, documented with the institution’s occupational medicine service, were excluded. Professionals selected for the sample were instructed to fill in the following printed forms at the end of their respective work shifts: PCV-3 referring to the set of tasks performed during work shift; a questionnaire with sociodemographic data; and a questionnaire with questions about general assessment and understanding of PCV-3 content. Data collection for the pre-test step took place between January and February 2022.

### Analysis of results, and statistics

The data obtained from all steps were recorded and organized in Microsoft Excel for Windows® spreadsheets, enabling a descriptive analysis of results. In the expert committee assessment step, the Content Validity Index (CVI) test was performed to assess the consensus between them. The minimum acceptable value for CVI was 0.80^( 20 )^. In the pre-test step, Cronbach’s alpha (α) test was performed to attest to the questionnaire reliability^( 21 )^, in addition to the CVI in the questionnaire on the general assessment and understanding of the translated version of NASA-TLX. Statistical tests were performed using the software Statistical Package for the Social Sciences (SPSS), version 26.0. In statistical analysis, the significance level adopted was 95%.

## RESULTS

The first three steps of cross-cultural adaptation and validity process of NASA-TLX took place with the help of companies certified in translating documents into English. Small discrepancies between synonymous words and degrees of adjectives used in sentence composition were sorted out by the main researchers.

In step IV, thirty experts were invited to compose the committee, however 14 accepted the invitation and answered the forms. Eleven experts were from southeastern Brazil (seven from the state of Sao Paulo and one from Minas Gerais); one from northern Brazil (state of Amazonas); one from midwestern Brazil (Federal District); and one was Brazilian who resided in the United States of America during the study period. Four were professionals with expertise in exact sciences (three from engineering and one from applied physics); three in humanities (literature and languages), with emphasis on English language; and seven in the biology field (five in nursing, one in nutrition and one in occupational therapy). One participant was a professor; eight held a PhD; and five held a master’s degree.

In the concordance analysis of NASA-TLX, translated version, items *“title and statement”, “performance” and “effort”,* in terms of semantic, idiomatic and conceptual assessments, did not reach the established minimum value of CVI=0.80 ( Table 1 ); therefore, they were reformulated and forwarded to the second step of expert committee assessment. Of the 14 experts participating in the first round of consensus, only seven participated in the second round of consensus, even after three attempts to contact them via email.

**Table 1:** Analysis of Content Validity Indices of the Portuguese consensus version - 2 (PCV-2), based on experts’ responses in the first consensus round, São Paulo, São Paulo, Brazil, 2022

	Semantics	Idiomatic	Cultural	Conceptual
*Title and statement*	0.714	0.714	0.786	0.714
*Mental demand*	0.929	1.000	0.929	0.929
*Physical demand*	0.929	1.000	0.929	0.857
*Temporal demand*	0.929	1.000	1.000	0.857
*Performance*	0.714	0.857	0.929	0.571
*Effort*	0.786	0.643	0.929	0.500
*Frustration*	0.929	0.929	1.000	0.929

In the item *“title and statement”* , regarding the title of the instrument in PCV-2, nine experts (64.28%) suggested adopting the term *“workload”* instead of *“task load”* . After analyzing this result, the main researchers met with two of the translators involved in the translation process and decided to keep the translation of the original term “task” to *“tarefa”* . As for the statement, after identifying inconsistencies between different versions of the tool, a consultation was carried out with the authors of the original scale, who reported that the utterance was not contemplated in the initial elaboration of NASA-TLX. Thus, it was decided to remove the statement in the version of NASA-TLX translated into Brazilian Portuguese, since it is not an instruction for completing the scale, and is therefore dispensable. These decisions and its justifications were presented to experts in the second Delphi round^( 19 )^, obtaining a consensus for both. Thus, the Brazilian Portuguese version maintained the title of the instrument as *“Índice NASA de carga de tarefa”* in PCV-3.

Still in the first step of analysis by an expert committee, a consensus was not reached regarding the terms used at the extremes of the response scale for item *“performance”* , being therefore changed from *“perfeito”* (perfect) and “ *fracasso* ” (failure), as proposed in PCV-2, to “ *muitíssimo* ” and “ *pouquíssimo* ” , maintaining the same format for the other five questions on the scale in PCV-3. Regarding the item “ *effort* ”, question consensus was not obtained, which was modified from “ *o quão difícil foi realizar a tarefa para atingir o seu nível de desempenho?* ” (as per version PCV-2) to “ *quanto esforço foi necessário para atingir o seu nível de desempenho no trabalho?* ”.

After consensus was obtained among experts for these items as well, PCV-3 was applied, as described in the pre-test step. Fifty health professionals from different categories were included. Participants’ sociodemographic characteristics are shown in Table 2 . As for participants’ responses to the translated version of PCV-3 into Portuguese, the averages obtained were: “mental demand” = 74.21; “physical demand” = 68.82; “temporal demand” = 71.96; “performance” = 24.31; “effort” = 73.82; “frustration” = 52.25.

**Table 2:** Sociodemographic characteristics of health professionals who participated in the pre-test step, São Paulo, São Paulo, Brazil, 2022

Characteristics	n	%
Sex		
Female	39	78
Male	11	22
Profession		
Nurse	12	24
Nursing technician	26	52
Nursing assistant	3	6
Doctor	5	10
Physiotherapist	4	8
Workplace		
Surgical ward	8	16
Medical clinic ward	10	20
Obstetrics and gynecology ward	6	12
Pediatric ward	8	16
Adult Intensive Care Unit	15	30
Several*	3	6
Professional experience		
Up to 1 year	4	8
1-5 years	29	58
6-10 years	10	20
11-20 years	6	12
More than 20 years	1	2
Work shift duration		
12 hours	44	88
11 hours	1	2
6 hours	3	6
24 hours	2	4
Work shift		
Day	28	56
Night	22	44

Several* - professionals who assisted different sectors in the same work shift.

The results of the general assessment of PCV-3 showed that 38 (76%) participants considered it good, nine (18%) fair, and three (6%) poor. Forty-three participants (86%) reported that all questions were easy to understand; 13 (24%) responded that some questions were difficult to understand; and 2 (4%) participants responded that all questions were difficult to understand. Regarding the understanding of the index’s response scale, 36 (72%) participants answered that it was easy; 12 (24%) considered regular understanding; and 2 (4%) considered it difficult. To validate content understanding of the six individual questions of PCV-3, CVI values obtained were greater than 0.80 in the pre-test step. The index’s internal consistency was confirmed using total Cronbach’s alpha result = 0.757. Table 3 shows CVI values for each item as well as Cronbach’s alpha scores, if an item was excluded.

**Table 3:** Content validity analysis and internal consistency of PCV-3 in the pre-test step, São Paulo, São Paulo, Brazil, 2022

	CVI	Cronbach’s α, if an item is excluded
Mental demand	0.900	0.727
Physical demand	0.900	0.694
Temporal demand	0.840	0.678
Performance	0.860	0.722
Effort	0.920	0.706
Frustration	0.860	0.727

*CVI - Content Validity Index.*

Regarding the suggestions and comments described by pretest participants, in general, participants highlighted that the tool measures the workload of a single work shift and that fluctuations in work demands can occur between shifts, influencing the index result. Additionally, it was commented that the questionnaire does not address teamwork influence on workload, and the inclusion of different feelings in a single question in “frustration” dimension can make it difficult to choose graduation in the response scale.

## DISCUSSION

This article aimed to describe the translation and cross-cultural adaptation process of NASA-TLX for workload assessment. After going through all the methodological steps, with rigor and according to the steps based on the literature, the intended results were achieved. It was obtained as a result that the consensus version in Brazilian Portuguese - 3 (PCV-3) is valid and reliable, being named “ *Índice NASA de carga de tarefa* ” (the file can be accessed through supplementary material).

As it is a rigorous method, which requires allocation of resources and efforts from researchers, the choice to conduct a tool’s translation and cross-cultural adaptation process should prioritize relevant tools that meet the objective for which they were created. The justification for choosing NASA-TLX was based on the fact that it is a short index, easy to apply and that can be used in different work contexts, not restricted to the health work field, in addition to being widely used in different countries^( 22 )^.

In the initial steps of the translation process carried out by professional translators, divergences were evident between the translation of specific terms, mainly when the translator was unaware of the objective of the index. Depending on the translated term, different interpretations can be made, varying according to each region’s cultural context. In this regard, the importance of including the “cultural equivalence” criterion in the expert committee assessment step in this study stands out. Some experts pointed out that the literal translation of terms “perfect” ( *perfeito* ) and “failure” ( *fracasso* ) from the original NASA-TLX “performance” item response scale could be misinterpreted by tool users. Therefore, these terms were replaced, maintaining the terms of the other items’ response scales: “ *pouquíssimo* ” and “ *muitíssimo* ” . In cross-cultural adaptation, this type of substitution facilitates scale interpretation, making the terms used more familiar and accessible to the target population^( 22 )^. Terms “good” and “poor” are also found in other versions of the scale^( 2 )^.

Given the regional and cultural differences in Brazil, in order to ensure the reliability of the translated and adapted version throughout the territory, professionals with different areas of expertise and from the five regions of the country were invited to compose the expert committee. However, professionals from only three regions of Brazil responded to the invitation and sent their responses, with a predominance of professionals living in the Southeast. However, digital technology use increased with the COVID-19 pandemic, facilitating contacts despite the physical distance, and the low return on responses was identified. The difficulty in having answers from experts is considered one of the disadvantages of the consensus Delphi technique^( 23 )^.

As for the title “ *Índice NASA de carga de tarefa* ”, no consensus was reached either regarding the use of “ *task load* ” instead of “ *workload* ”. In the final version, a consensus was reached on maintaining the translation of the original term “task” into “ *tarefa* ”, considering that the questionnaire could be applied both in relation to a specific task and to a set of tasks after a work shift, as performed in the pre-test step of this study. The use of NASA-TLX was validated to assess the load of a set of tasks through a study involving adults with type 1 diabetes in the United States, after performing a set of self-care activities^( 24 )^.

In another study, NASA-TLX was also used to assess the burden of a set of tasks among nurses working in Critical Care Units after their work shifts^( 25 )^. Therefore, there are subsidies for the application of NASA-TLX translated into Brazilian Portuguese after performing a specific task or a set of tasks, and it is also possible to adopt the term “workload” ( *carga de trabalho* ). However, it is suggested that, when applying NASA-TLX after a set of tasks or a work shift, researchers consider that different factors may influence the results that will be obtained. It is likely that workload is not an isolated element, but understood in conjunction with other activities in a given period. Therefore, it is desirable that additional studies be carried out analyzing whether there are differences in the accuracy of NASA-TLX in the specific use of a given task or in the set of tasks throughout a work shift.

In the pre-test step, more than 70% of participating health professionals assessed that, in general, the validated Brazilian Portuguese version of NASA-TLX was good and that both the questions included in the index and the response scale were easy to understand. Additionally, reliability and consistency were attested through CVI scores above 0.80 and Cronbach’s alpha above 0.70. These data corroborate that NASA-TLX Brazilian Portuguese version is useful for assessing workload in the context of health care, especially in the face of health crises, such as the COVID-19 pandemic, which can influence quality of care^( 26 )^.

Over the decades since its elaboration, the analysis of NASA-TLX scores has undergone adaptations, and one of them consists of using each domain’s raw scores, in addition to a general score to estimate the global task load through the sum of individual scores. Previously, the authors recommended assigning weights to each domain, according to the number of times each of them was rated as significant by participants. However, this process has been considered complex and ineffective. The use of raw scores, as performed in the pre-test of this study, and frequently adopted in other studies, facilitates applying the instrument and makes it possible to compare each domain’s individual scores, providing the inference of which domain exerts greater or lesser influence on task load^( 26 , 27 )^.

Shoja et al. showed, through the application of NASA-TLX among different health professionals responsible for caring for patients with COVID-19, that nurses reported higher task load scores, and the “mental demand” dimension was the one that presented the highest scores^( 28 )^. In the pre-test step, the “mental demand” dimension was also the one with the highest average score. However, as this is a study of cross-cultural adaptation and validity in a restricted sample, it is not possible to generalize the results obtained from the application of the scale.

### Study limitations

This study has some limitations. The low response rate, with the participation of three out of the five Brazilian regions, may have limited the cultural contextualization of this translation. Moreover, the translation carried out was made into Brazilian Portuguese and may not fully correspond to the Portuguese of other countries. Carrying out the pre-test step in a single health service allowed analysis within a specific scenario, which may not necessarily correspond to other similar scenarios. However, these limitations do not compromise the result of the cross-cultural adaptation process, content validity and reliability of NASA-TLX, which is presented as an instrument available for immediate use in Brazil.

### Contributions to the areas of nursing and health

In the health field, bearing in mind future research prospects, NASA-TLX Brazilian Portuguese version can also be used to compare different work devices, such as new PPE, and even in the implementation of new technologies for work processes, such as computer softwares. Additionally, this index may serve as a parameter for comparing task load, helping in the decision-making process of planning and allocating work teams. New studies may also expand the psychometric properties of the index applicable to other types of work activities.

## CONCLUSIONS

The translated instrument “ *Índice NASA de carga de tarefa* ”, Brazilian Portuguese version, presents reliability and content validity evidence. Although the original instrument had few items, it was identified that the translations of some terms could be subject to misinterpretation, compromising the reliability of the instrument. After the adaptation steps, these flaws were minimized, resulting in a tool that can be used in different work settings. It is suggested that new psychometric studies, using the index presented in this article, be carried out in other work contexts in the Brazilian territory.
